# A qualitative, mixed-method approach to reaching consensus on function, fatigue, and fatigability outcomes in teens and adults living with spinal muscular atrophy

**DOI:** 10.1186/s13023-025-04047-x

**Published:** 2025-10-17

**Authors:** Jacqueline Glascock, Lisa T. Belter, Meghan Moore Burk, Jessica J. Tingey, Mary A. Curry

**Affiliations:** 1 Present Address: Cure SMA, 925 Busse Road, Elk Grove Village, IL 60007 USA; 2 Children’s Hospital Colorado, 13123 East 16th Avenue, Aurora, CO 80007 USA

**Keywords:** Activities of daily living, ADLs, Clinician-reported outcome measure, CROM, Perceived fatigue, Perceived fatigability, Patient-reported outcome measure, PROM, Spinal muscular atrophy, SMA

## Abstract

**Background:**

Spinal muscular atrophy (SMA) is a neuromuscular disease caused by mutations in the survival motor neuron gene, *SMN1*. Loss of *SMN1* function results in deficiency of the SMN protein leading to motor neuron death, muscle wasting, and progressive loss of motor function. Two disease modifying therapies have been approved for teens and adults in the United States, with many more potential treatments in the drug development pipeline. As treatment options for teens and adults with SMA increase, a validated core set of outcome measures is needed to assess motor function, perceived fatigue, and perceived fatigability. The aim of this study was to determine which type of outcome measures best captures changes in disease status in teens and adults with SMA.

**Results:**

In the first phase of this two-part study, a working group of key opinion leaders in SMA research and clinical care was surveyed using a modified Delphi method. The working group concluded that a patient-reported outcome measure based on activities of daily living (ADLs) would be the best way to capture changes in function, perceived fatigue, and perceived fatigability that are meaningful to clinicians, as well as teens and adults living with SMA. In the second phase of the study, two discussion groups of adults (non-ambulatory or ambulatory) were interviewed for their perspectives about which ADLs are most important to them, and about how perceived fatigue affects their abilities to perform these ADLs. Both discussion groups prioritized ADLs that related to independence and dignity. Non-ambulatory and ambulatory participants also reported that perceived fatigue and fatigability are a major factor in their ability to perform ADLs.

**Conclusion:**

SMA key opinion leaders and adults with SMA agreed that ADLs would be sensitive and impactful outcomes in the assessment of function, perceived fatigue, and perceived fatigability. The findings of this study form a foundation for reaching consensus around a core set of outcome measures for assessing disease status, perceived fatigue, and perceived fatigability in teens and adults with SMA in the U.S.

## Background

### Spinal muscular atrophy (SMA)

Spinal muscular atrophy (SMA) is an autosomal recessive disease that was, until recently, the leading genetic cause of mortality in children under the age of two years [1–3]. In 95% of cases, SMA is caused by a biallelic mutation in the survival motor neuron 1 gene (*SMN1*) [4–6]. Mutation of the *SMN1* gene results in deficiency of the survival motor neuron protein (SMN), which has a variety of functions throughout the body and is especially important in the brainstem and spinal cord [7–9]. Untreated SMA is characterized by progressive motor neuron loss that may result in limb and truncal muscle weakness, scoliosis, contractures, and respiratory complications [10–19]. In the most severe forms of SMA, symptoms can be present at birth and progress rapidly, and early diagnosis and treatment with a disease modifying therapy (DMT) are necessary to achieve optimal outcomes [20–23]. With less severe types of SMA, symptom onset may occur later in life, and loss of function may be more gradual [12, 24–28]. Like *SMN1*, the survival motor neuron 2 gene (*SMN2)* also encodes the SMN protein. Although much of the protein produced by *SMN2* is truncated and dysfunctional, *SMN2* can act as a partial “back-up” for the mutated *SMN1* gene in people with SMA. As such, *SMN2* serves as a critical disease modifying gene [29, 30]. 

SMA phenotype has historically been classified into five types, 0–4, which are determined by the age of symptom onset and maximum motor function achieved [11, 12, 30, 31]. However, the recent availability of DMTs for SMA has dramatically impacted the clinical course of the disease and led to a shift in classification based on *SMN2* copy number and motor function [32]. In 2016, the United States (US.) Food and Drug Administration (FDA) approved the antisense oligonucleotide, *nusinersen*, for use in children and adults with SMA [33]. Next, a gene therapy, *onasemnogene abeparvovec*, was approved by the FDA in 2019 for use in children under the age of two [34]. Finally, the RNA splicing modifier, *risdiplam*, was approved by the FDA for patients older than 2 months in 2020 and for patients of any age in 2022 [35]. Each of these “SMN-dependent” therapies works by restoring SMN protein levels, which protects motor neurons and preserves muscle function [20–23]. However, due to disease phenotype heterogeneity, and variability in disease onset and the timing of therapeutic intervention, treatment response varies widely. Therefore, critical treatment needs remain unmet within the SMA community. For example, teens and adults with SMA who do not receive DMT until later in their disease progression may experience less dramatic benefits from treatment [36–38]. To meet remaining treatment needs, the SMA drug pipeline is burgeoning with studies of new potential, SMN-independent treatments, as well as novel protocols for approved SMN-dependent treatments [39]. In the future, it is likely that a combination of SMN-dependent and SMN-independent therapies will be used to achieve optimal patient outcomes [40–42]. 

### Clinical outcome measures for SMA

A clinical outcome measure is an endpoint that assesses how disease progression and treatment affect how a patient feels, functions, or survives [43]. Clinical outcome measures may be clinician-reported (CROM) or patient-reported (PROM). Myriad CROMs and PROMs have been utilized in SMA research and clinical care to assess functional abilities. For example, a literature review and clinician survey performed by Slayter et al. in 2021 [44] identified 17 different CROMs that had been used or were still in use internationally in the evaluation of adults with SMA. Similarly, a recent scoping review of PROMs for SMA determined that between 2016 and 2022, 31 unique PROMs were utilized in studies conducted internationally with adults living with SMA and other neuromuscular conditions [45]. Of these 31 PROMs, few included a comprehensive set of health domains, and only two were developed explicitly for people with SMA. In addition to changes in motor function, fatigue and fatigability are often reported as major concerns by teens and adults with SMA [46–48]. Although the nomenclature for fatigue and fatigability has varied [48–51], the emerging trend is to separate the concepts into two subtypes: performance (clinician-reported) fatigability and perceived (patient-reported) fatigue and fatigability [50, 51]. Performance fatigability is an objectively quantified lack of endurance, which manifests as the diminished ability to complete or repeat a specific physical task within a given time frame [52]. Perceived fatigue is a person’s subjective feeling of exhaustion, which may have physical, cognitive/intellectual, and emotional/social components. Also subjective, perceived fatigability is a person’s estimation of their susceptibility to exhaustion that occurs while performing a task of a specific intensity and duration [51, 53]. The Fatigue Severity Scale (FSS) [54], Multidimensional Fatigue Inventory (MFI) [55], Spinal Muscular Atrophy Health Index (SMA-HI) fatigue subscale [56], PROfuture scale [57], and SMA-TOOL scale [58] have been validated for assessing perceived fatigue in people with SMA. However, some research suggests these measures are not sensitive enough to capture how perceived fatigue impacts the daily lives of people with SMA [51, 59]. A scale for assessing perceived fatigability has not yet been validated for use in SMA research and care, but such work is currently underway [53]. 

It is estimated that in the U.S., approximately 60% of people with SMA are teens or adults [60]. As more teens and adults with a broad range of SMA phenotypes receive investigational and FDA-approved treatments in the U.S., a standardized set of clinical outcomes is needed to measure changes in motor function, perceived fatigue, and perceived fatigability in teens and adults. The establishment of core clinical outcomes for SMA would facilitate comparisons of research data across studies and provide a consistent standard of care across the nation [61]. 

Cure SMA is the largest SMA patient advocacy organization based in the U.S. that funds and directs research for SMA [62]. In this study, Cure SMA convened (1) a working group of key opinion leaders (KOL Working Group) in SMA, comprising researchers, health care providers, and biotechnology industry representatives, and (2) two discussion groups comprising either non-ambulatory adults (Non-ambulatory Discussion Group) or ambulatory adults (Ambulatory Discussion Group) living with SMA. The aim of the study was to build consensus on which clinical outcomes best capture changes in disease status and motor function, as well as perceived fatigue and fatigability, in teens and adults with SMA in the U.S.

## Methods

### Overview

**Fig. 1:**
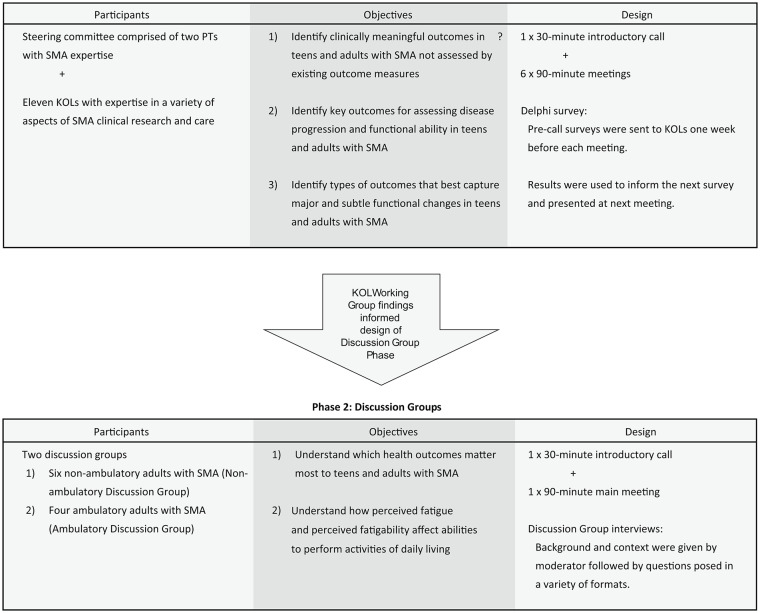
Components of the two-phase, mixed-methods study. KOL = key opinion leader

A two-phase, mixed-methods study was utilized to build consensus around which are the most valuable CROMs for teens and adults with SMA. (Fig. 1) In the planning stages of the study, two physical therapists (PTs) with expertise in SMA were consulted on study design and content. These PTs also participated as a Steering Committee in the KOL Working Group phase of the study. A third-party consultant, The Kith Collective, coordinated, organized, and conducted Discussion Group meetings with guidance from Cure SMA. PTs and Kith Collective employees were compensated for their time, KOLs participated as volunteers, and Discussion Group participants received a gift card ($50) for their participation.

### Phase 1: KOL Working Group

KOL Working Group objectives were to (1) identify clinically meaningful outcomes in teens and adults with SMA not assessed by existing outcome measures, (2) identify key outcomes within core functional domains for assessing disease progression and functional ability in teens and adults with SMA, and (3) identify types of outcomes that best capture major and subtle functional changes in teens and adults with SMA. The KOL Working Group was composed of two Steering Committee members and 12 KOLs. KOLs were invited by email to participate based on their expertise in SMA clinical care and research. (Additional File 1) For logistical simplicity, invitations were restricted to KOLs residing in the U.S. A modified Delphi method was utilized to achieve consensus. The process consisted of a 30-minute kick-off call on February 9, 2022, followed by six, 90-minute KOL Working Group meetings occurring between March 9, 2022, and October 11, 2022; all meetings were held over Zoom. To prepare the KOLs for discussions, peer-reviewed articles were gathered by Cure SMA staff on the following topics as they relate to outcome measures for SMA: bulbar function, fatigue and fatigability, activity limitations/disease burden, respiratory function, and general function. At the kick-off call, Cure SMA distributed briefing packets containing the background reading. Before each of the remaining six KOL Working Group meetings, the participants completed a 5-minute, anonymous, multiple choice/open response survey. (See Additional File 2 for all Working Group surveys.) Pre-call surveys were sent one week prior to the next meeting and were to be completed by the day before the next meeting.

Cure SMA staff reviewed and distilled survey results between meetings, then presented and discussed findings with the KOLs at the following meeting. Survey results were also utilized by Cure SMA to generate a new survey in advance of the next meeting to work toward consensus on core outcomes. Each Zoom meeting was recorded, and minutes were distributed by email the following week. At the conclusion of Phase 1 of the study, key questions that emerged during the Delphi process were identified by Cure SMA and The Kith Collective. These questions informed the discussion guide (Additional File 3) for the next phase of the study.

### Discussion Group

Discussion Group objectives were (1) to understand which health outcomes matter most to adults with SMA, and (2) to understand how perceived fatigue and perceived fatigability affect abilities to perform activities of daily living (ADLs). An eligibility survey (Additional File 4) was created and shared via email with teens and adults with SMA that reside in the U.S. as identified through the Cure SMA Membership Database [63]. Eligibility survey responses were received from 261 individuals and were organized into a perspectives matrix created by Cure SMA staff. Responses to demographic and patient experience questions were used to facilitate diverse enrollment. (Additional File 5) Twenty-three persons with SMA were invited to participate in the discussion groups; informed consent was obtained from 10 individuals. Participants were divided into two discussion groups based upon whether the individual was non-ambulatory or ambulatory. The Non-ambulatory Discussion Group contained six adults with SMA, and the Ambulatory Discussion Group four adults with SMA. (Additional File 6) Due to limited response to recruitment, neither discussion sub-group contained teen participants.

For each Discussion Group, a 30-minute introductory meeting was held, followed by a 90-minute main meeting. All meetings, led by the Kith Collective, were held over Zoom and recorded. During the introduction meeting, the background and intent of the study was described, and participants were introduced to the concept of ADLs. In advance of the main meeting, participants were asked to consider a list of sample ADLs and to think about other activities that were important to them. (Additional File 7) This exercise was meant to prepare participants for upcoming discussions in the main meeting. Participants were also introduced to the concepts of physical, cognitive/intellectual, and emotional/social fatigue. (The delineation between perceived fatigue and perceived fatigability had not yet been formalized within the field of SMA research at the time of the study.)

Prior to initiation, WIRB-Copernicus Group Institutional Review Board (WCG IRB) reviewed and approved the study (IRB Tracking Number: 20226344). All respondents were informed via the informed consent document that findings may be published. Only de-identified results are included within the manuscript. All procedures performed involving human participants were in accordance with the ethical standards of the institutional and/or national research committee and with the 1964 Helsinki declaration and its later amendments or comparable ethical standards. During the sessions, notes were taken by Kith Collective and Cure SMA staff and used to identify themes in participant feedback, as was chat feedback from those participants that elected to provide written responses.

## Results

### Phase 1: KOL Working Group

Through reiterative surveys and discussions, KOL Working Group participants worked toward consensus on the most meaningful outcomes for measuring function and disease status in teens and adults with SMA. First, KOL Working Group participants identified clinically relevant outcomes, within each of five domains, that were not captured by existing outcome measures for non-ambulatory and ambulatory teens and adults with SMA. (Table 1) As the group discussed these outcomes, two themes emerged: (1) ADLs may be utilized as meaningful outcomes for assessing function in non-ambulatory and ambulatory teens and adults with SMA, and (2) perceived fatigue and perceived fatigability are major aspects of SMA that may affect abilities to perform ADLs.

**Table 1:** Domains and outcomes not captured by existing outcome measures for teens and adults with SMA

Domain	Outcome
Bulbar function (encompassing swallow, voice, vocal capacity, phonation, speech)	• Ability to capture slow disease progression/early deficits/incremental changes/increased sensitivity to longitudinal change • Jaw range of motion • Tongue strength • Bite strength • Chewing fatigue/endurance of chewing • Ability to be understood when speaking • Ability to maintain good nutrition • Risk of aspiration • Swallowing
Perceived fatigue and perceived fatigability^a^	• Ability to complete meals, time to finish meals • Functional metrics/current tools have reduced carryover to true functional activities • Ability to function for entire day • Assessment of sleep • Respiratory function
Cognition	• Neurodevelopmental scores/neurophysiological evaluation • School readiness functions • Prospective natural history or evaluation of cognition in teens/adults is an area of need
Gross motor function/range of motion/strength	• Ability to self-transfer, nocturnal rotation • Wheelchair-based assessments (i.e., ATEND) • Impact on daily function • Ability to manage assistive devices
Fine motor function	• Functions related to use of social media/computer tasks/communication (typing, texting, etc.) • Ability to manage assistive devices (wheelchair joystick) • Ability to feed self • Grasp and grip

Survey 1 results (*n* = 9): KOL Working Group participants were asked, “When thinking about outcomes for teens and adults with SMA, which outcomes/items in [domain] not captured by currently existing outcomes do you feel would be clinically meaningful?”

^a^The terms “fatigue and fatigability,” which were used in the original study, have been changed to “perceived fatigue and perceived fatigability” to reflect current operational definitions

### Activities of daily living (ADLs)

As discussions unfolded, KOL Working Group participants agreed on the need for specific, finite outcomes that are sensitive enough to detect both major and minor changes in function. The group reasoned that teens and adults with SMA are likely to experience subtle changes in function over time, whether from disease progression or treatment, and these subtle changes may profoundly impact participation in daily activities and quality of life. Consensus began to develop that ADLs were likely to be the most meaningful type of outcome for teens and adults with SMA because these age groups tend to describe differences in disease status (i.e., improvements or reductions in function) through changes in their abilities to complete everyday tasks. KOL Working Group participants were asked to rank outcomes within five domains in terms of which would be most clinically meaningful in assessing teen and adult functional status. (Table 2) In discussion, KOL Working Group participants also noted that transfers, socialization and communication, transportation, toileting, and the ability to move outside the home were additional important domains for both non-ambulatory and ambulatory people with SMA.

**Table 2:** KOL Working Group key outcomes for assessing functional ability in teens and adults with SMA

Domain (number of respondents)	Outcomes	Overall Rank	Score	No. of Rankings
Bulbar Function (*n* = 6)			(max score = 30)	
	Risk of aspiration	1	22	6
	Swallowing/dysphagia	2	19	5
	Ability to maintain good nutrition	3	16	5
Perceived Fatigue (*n* = 5)			(max score = 25)	
	Time on ventilation	1	13	4
	Ability to complete age-appropriate play/specific tasks during the day	2	12	3
	How long can one perform a task before fatigue limits it	3	11	4
Performance Fatigability^a^ (*n* = 5)			(max score = 25)	
	Ability to function for entire day	1	24	5
	Ability to complete meals/time to finish meals	2	16	4
	Ability to drive power chair	3	8	3
Cognition/Language/Communication (*n* = 4)			(max score = 20)	
	Ability to communicate	1	20	4
	Intelligibility (especially, if muscle weakness is impeding intelligibility)	2	10	3
	School assessments/school readiness	3	8	3
Gross Motor Function/ Range of Motion/Strength (*n* = 5)			(max score = 25)	
	Ability to manage assistive devices (wheelchair joystick)	1	18	5
	Functions related to use of social media/computer tasks/communication (typing, texting, etc.)	2	15	5
	Strength to make transfers outside of home	3	10	3
Fine Motor Function (*n* = 5)			(max score = 25)	
	Ability to manage assistive devices (wheelchair joystick)	1	22	5
	Functions related to use of social media/computer tasks/communication (typing, texting, etc.)	2	21	5
	Ability to feed self	3	8	2

Survey 2 results: KOL Working Group participants were asked to rank up to five outcomes in each domain according to clinical relevance. The three highest-ranked patient outcomes in each domain are shown in this table. Outcomes were given a value of 1 through 5 based on ranking, with the highest-ranked outcomes given a value of 5 and the lowest a value of 1. Therefore, the maximum possible score (max score) for each outcome was 5 times the number of respondents who ranked that outcome. ADLs = activities of daily living; KOL Working Group = key opinion leader working group

^a^The original terminology used in the study was, “functional (performance) fatigue.” Terminology has been updated to align with the current operational definition, “performance fatigability”

KOL Working Group participants then weighed the utility of ADLs against other types of outcomes used to assess function in teens and adults with SMA. (Table 3) KOL Working Group participants concluded that ADLs may be leveraged to simultaneously assess gross motor functions and detect minor functional changes that might be missed by other types of outcomes. Group participants noted that using an outcome measure based on ADLs alongside a CROM and/or a biomarker [64–66] was likely to give the most broad and detailed information about disease status in teens and adults with SMA.

**Table 3:** Outcome types ranked by ability to capture functional status in teens and adults with SMA

Survey 3 results (*n* = 3)			
Outcome Type	Overall rank	Score (max = 15)	No. of Rankings
Activities of daily living (ADLs)	1	15	3
Gross motor milestones	2	11	3
Write-in responses^a^	3	9	3
Compound muscle action potential (CMAP)	4	5	2
Other biomarker	5	1	1

Survey 4 results (*n* = 7)			
Outcome Type	Overall rank	Score (max = 28)	No. of Rankings
Gross motor milestones	1	28	7
Activities of daily living (ADLs)	2	28	6
Compound muscle action potential (CMAP)	3	20	7
Write-in responses^b^	4	13	6
Other biomarker^c^	5	10	6

In Surveys 3 and 4, KOL Working Group participants were asked to rank types of outcomes in terms of their utility in assessing functional status in teens and adults with SMA. Survey 3 results: ^a^Write-in responses: The Moore Scale (Moore Scale = EVOLVE-SMA [67]); respiratory function; access to needed equipment; time spent on medical/therapeutic interventions; if time for care interferes with other desired activities. Survey 4 results: ^b^Write-in responses: specific evaluations that assess ADLs and gross motor milestones; HFMSE, RULM scores, and 6MWT; patient reported measures of participation (school, work, community), social function; mouth opening; assessments which might be informative would be use/change of respiratory supports; changes in PT/OT status and need for additional supports (wheelchair, etc.); change in need for nutritional support; new surgeries (progressive scoliosis, etc.). ^c^Other biomarker response: MUNE. Outcomes were given a value of 1 through 5 based on ranking, with the highest-ranked outcomes given a value of 5 and the lowest a value of 1. Therefore, the maximum possible score (max score) for each outcome was 5 times the number of respondents who ranked that outcome

ADLs = activities of daily living; 6MWT = Six Minute Walk Test; HFMSE = Hammersmith Functional Motor Scale Expanded; MUNE = motor unit number estimation; OT = occupational therapist; PT = physical therapist; RULM = Revised Upper Limb Module

**Fig. 2:**
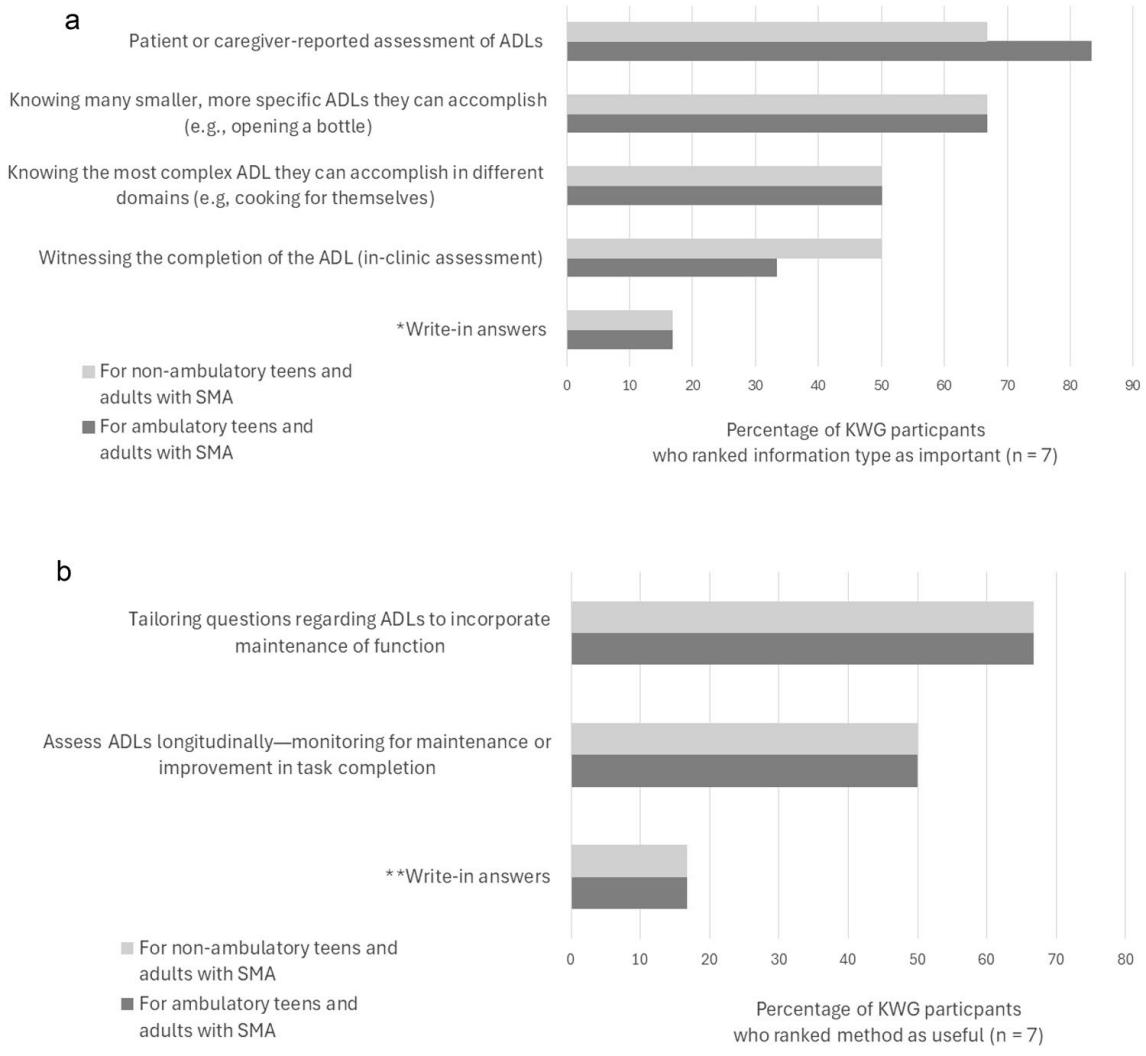
**a** KOL Working Group participants indicated which information would best aid in assessing disease status. *Write-in responses for non-ambulatory and ambulatory patients were: “Surrogate markers of function needed to complete ADLs” and “Would also note improvement in fatigue/fatigability,” respectively. **b** KOL Working Group participants indicated which method would best aid in assessing disease status. *Write-in comments for non-ambulatory and ambulatory patients were “Would also monitor fatigue and fatigability " and “Would also note improvement in fatigue and fatigability,” respectively

Having determined that ADLs were potentially the most useful type of outcomes for assessing function in teens and adults with SMA, KOL Working Group participants discussed the most effective methods for gathering information on individuals’ ability to perform these tasks. For both non-ambulatory and ambulatory individuals with SMA, a majority of group members agreed that information would be most clinically meaningful if it was reported by the patient or their caregiver, and if it described smaller tasks required for specific ADLs. (Fig. 2a) A majority of KOL Working Group participants also indicated that questions about ADLs should be tailored to detect changes in function over time for both non-ambulatory and ambulatory teens and adults with SMA. (Fig. 2b) In discussion, the group agreed that when asking teen and adult patients with SMA about ADLs, it would be important to note if the patient requires assistive technology, a specialized environment, or the assistance of another person to achieve the activity.

### Perceived fatigue and fatigability

**Fig. 3:**
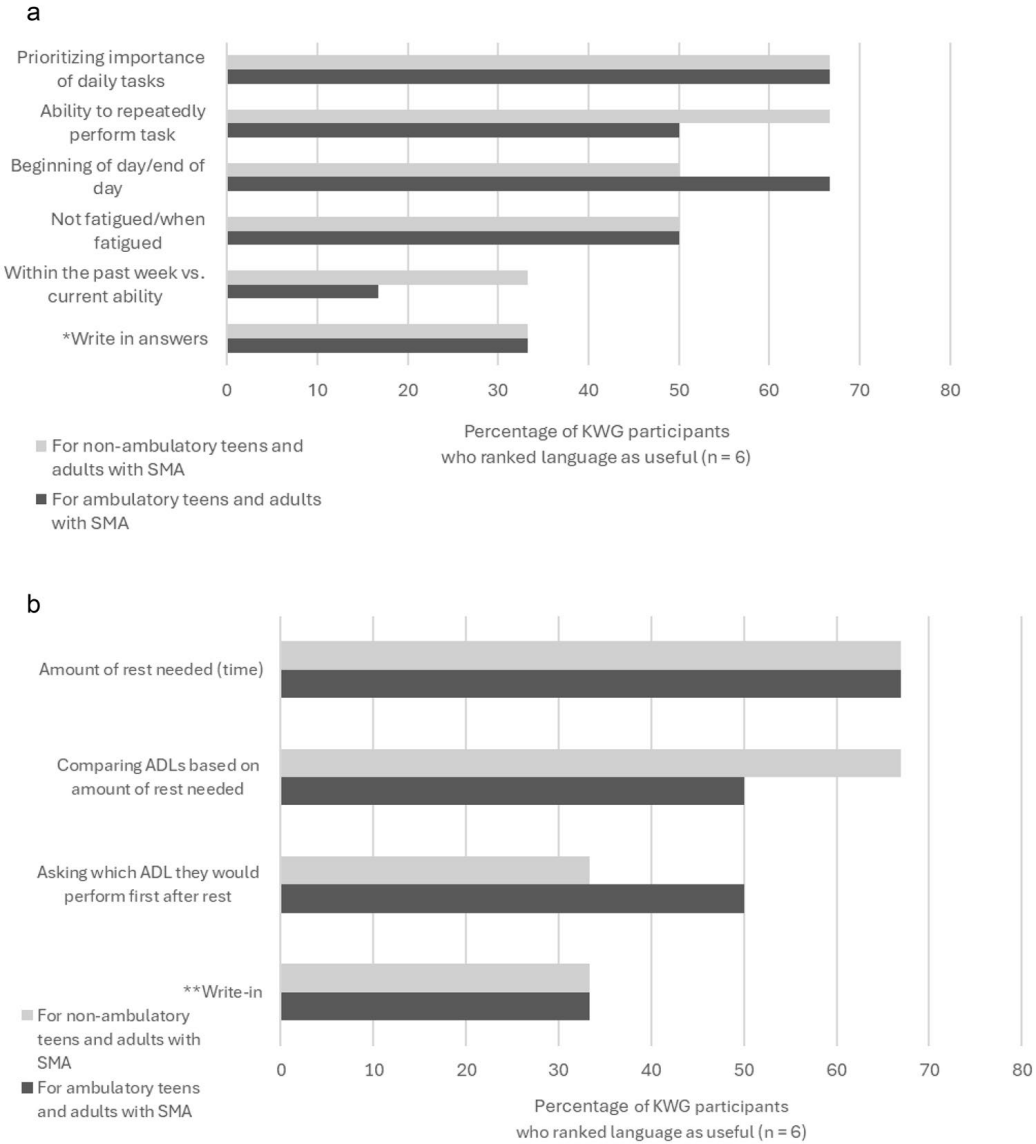
**a** KOL Working Group participants indicated which language would be most useful in asking how fatigue affects ADLs. *Write-in answers for both non-ambulatory and ambulatory patients with SMA: “Might ask about task becoming too difficult or taking more time to complete” and “Prioritization of tasks and fatigability later in the day may be the most relevant; but others are of interest as well.” **b** KOL Working Group participants indicated which language would be most useful in assessing changes in recovery time needed after heavy exertion. **Write-in answer for both non-ambulatory and ambulatory patients with SMA: “When you are overly fatigued, how much rest (in hours) is typically required before you can resume your typical daily activities?”

As Phase 1 of the study progressed, KOL Working Group participants repeatedly remarked that perceived fatigue and fatigability are major issues with non-ambulatory and ambulatory teens and adults with SMA, and that these phenomena are difficult to describe and measure. The KOL Working Group discussed existing outcome measures of perceived fatigue and fatigability, noting that they were not aware of any that had been validated for SMA. The KOL Working Group concurred that ADLs could be utilized as outcomes to measure changes in perceived fatigue and fatigability. A majority of KOL Working Group participants indicated that an ADL-based outcome measure should use a variety of questions when querying patients about changes in daily perceived fatigue and fatigability. (Fig. 3a) KOL Working Group participants noted that both non-ambulatory and ambulatory patients with SMA report a “refractory period” of perceived fatigue after heavy exertion. When the group ranked methods of assessing this refractory period, a majority preferred measuring the amount of rest time needed after overexertion for both non-ambulatory and ambulatory patients with SMA. For non-ambulatory patients with SMA, a majority also favored comparing ADLs based on amount of rest time needed. (Fig. 3b)

### Discussion Group questions

Phase 1 results of this study yielded consensus among KOL Working Group participants that ADLs may be utilized as outcomes to assess function, perceived fatigue, and perceived fatigability in non-ambulatory and ambulatory teens and adults with SMA. KOL Working Group participants agreed that gathering input from individuals with SMA was an essential next step in developing a core list of ADLs. Using Phase 1 data and feedback, Cure SMA developed the following questions around which to build discussion guides for the patient focus groups surveyed in Phase 2.

1. In addition to the sample ADL domains discussed by the KOL Working Group, would Discussion Group participants like any additional domains included in an outcome measure?
2. Which ADLs would Discussion Group participants most like to have stabilized? e.g., “I want to make sure I have enough strength to continue to use the bathroom independently.”
3. What do Discussion Group participants feel would be the best way to show/tell their provider about their fatigue/changes in fatigue? e.g., “What do you wish your doctor knew about your fatigue?”

### Phase 2: Discussion Group

#### Non-ambulatory Discussion Group

##### Activities of daily living (ADLs)

The first objective of the Non-ambulatory Discussion Group was to obtain feedback from participants about which ADLs were most important to them. Non-ambulatory Discussion Group participants were asked to review a list of ADLs (Additional File 7) and indicate other ADLs of importance that were not included. Transitioning between indoors and outdoors, caring for and interacting with pets, and using one’s voice were noted as key ADLs by Non-ambulatory Discussion Group participants. Many aspects of voice use were discussed by the subgroup. Participants explained how, in addition to facilitating communication, their voices play important roles in using verbal commands to operate items ranging from a light switch to a power wheelchair.

I do everything with my voice.

As the group discussed ADLs that were meaningful to them, the need to customize ADLs to the person being assessed became a major theme. Group participants noted that the abilities of adults to perform ADLs varies widely depending on age, stage of disease progression, and physical accommodations.

A second theme emerged as several group participants noted that many of the most important ADLs to them were those that fell beyond self-care and household chores and extended into work, relationships, hobbies, and other forms of self-expression.

I don’t see any work, I don’t see any recreation, I don’t see any going out in the community or anything like that. And to me that’s all more part of a day than all these things. All these things are part of everyday, but the list here sounds like we get dressed, we take a pill, we eat something, and that’s all we do all day…There’s a lot more in there with that.

For me, what’s most important is art and music. I play instruments and do art, and so losing that would really affect my quality of life.

This is kind of unusual, but it is important to me, and I kind of made it a category of its own called ‘emotional.’ Like hugging people, I miss not being able to physically hug people. Physically interacting with people.

The group also concurred that abilities to perform ADLs can vary from day to day and are influenced by environmental factors like cold, which impacts fine motor skills.

Drawing/painting/3D printing. Tasks of repair and maintenance that might be simple for an able-bodied person—some days I can do them and some days not.

All those tasks are simple for an able-bodied person, but for someone with a disability—sometimes I can do it on my own, and sometimes I need assistance, depending on my ability level that day.

Many NGD participants had already lost abilities to do more routine ADLs independently and relied on help from others or assistive devices to perform self-care and household chores. As such, getting access to help from (family or paid) caregivers was itself an essential ADL.

As long as the proper supports are there, it is probably what I fret about most, is losing my ton of support.

I think that’s really the key—it’s caregivers and access to caregivers—because we could make a list five times this length, but at the end of the day, as long as you have reliable caregivers and access to help a lot of these things don’t matter as much.

However, other Non-ambulatory Discussion Group participants considered needing assistance to complete an ADL an extra burden. One participant explained it was frustrating to have to wait for 5 to 30 minutes for help to arrive before they could accomplish a task.

A fourth theme that formed was the desire to maintain independence and dignity. Many of the ADLs that were most important to group participants fell into the domains of maintaining one’s own comfort, health, hygiene, and safety. One Non-ambulatory Discussion Group participant said that retaining the ability to make their own healthcare decisions was very important to them. Another said they feared losing the ability to breath on their own, because the loss would entail “so much more dependence and complications.”

…This is kind of embarrassing, but crying, being able to wipe your own face and blow your nose, it’s embarrassing, but it’s important.

The group was then asked by the moderator, “How hard or easy do you think it would be to measure your overall health status based on your ability to perform ADLs over the course of time?” Non-ambulatory Discussion Group participants agreed that changes in ability to perform ADLs were more likely to reflect changes in function than in overall health status. Several participants explained that they thought of their overall health status as independent from SMA.

It all goes back to how we see ourselves as normal. From the perspective of medical professionals, we are unhealthy…What do you mean by healthy? Because, we don’t think we’re not healthy. We’ve embraced our disability.

Just because I can’t feed myself anymore, I don’t think I’m less healthy. Being able to type on a keyboard, I don’t see that as a measure of my health.

Non-ambulatory Discussion Group participants added that although abilities will vary day to day, these variations may not reflect a change in overall health status.

Non-ambulatory Discussion Group participants were shown an example of the type of scale that may be used in a questionnaire asking about ADLs. The scale included the following rating options: Unable – can’t do, doesn’t know how, or is too young; Hard – does with a lot of help, extra time or effort; A little hard – does with a little help, extra time, or effort; and Easy – does with not help, extra time or effort, or a person’s skills are past this level. The moderator asked, “If you are asked to rate your ability to perform various ADLs using a scale like this, would you find it easier to rate the broad domain like ‘feeding oneself’ or would you want to be able to break that task into different parts?” The majority agreed that asking detailed questions in which ADLs had been broken down into smaller tasks would likely produce more accurate answers because people with SMA have different adaptive strategies.

We can do a lot of this stuff but will probably do it a little differently. For example, feeding ourselves, you know, some of us can pick it up and just go for it, others may have to hold the fork a different way, or you know, use a different utensil, use both arms—so I think breaking it up would be the way to go.

You can make a phone call, but can you get a phone? That’s why being specific is important.

A third group participant noted that breaking ADLs into smaller tasks would clarify whether the respondent was able to perform the ADL independently or required assistance. They extrapolated,

When I hear these questions coming from a perspective, like a medical research perspective, I assume they want to know, can I do these things myself? And the answer to that is, “No.” But can I do them? Well, yes, I do them every day, with peoples’ help. So, the way I judge it is, “Can you do it with someone’s help?” And I would answer, “Yes.” But I would think I would give you a wrong idea of what I can do.

However, one group participant thought that broader ADLs domains would be easier to rate. They explained,

My life is pretty defined by what I can do by myself and what I can’t do by myself, so this makes it a little easier to kind of categorize it in my brain.

**Table 4:** Summary of feedback from Non-ambulatory Discussion Group participants*

Topic	Major theme	Subtheme
ADLs	ADLs should be customized to the individual.	People with SMA have different ranges of abilities. People with SMA have different lifestyles and priorities.
	ADLs should encompass activities beyond self-care and household chores.	Work, hobbies, relationships, and interacting with the community are all important domains.
	Abilities to perform ADLs can vary from day to day and in different situations. Preserving abilities to perform ADLs that enable independence and dignity is critical. ADLs should be utilized to assess a person’s functional status rather than overall health. ADL assessments should ask detailed questions.	Abilities to perform ADLs may sometimes depend on assistive devices, environmental adaptations, or help from a family member or caregiver. Maintaining one’s own comfort, health, hygiene, and safety are key to preserving independence and dignity. Some people with SMA may think of their overall health as separate from SMA. Some people with SMA may consider themselves disabled but not unhealthy. People with SMA may have different ways of accomplishing the same task, which would be revealed by more detailed questions.
Perceived fatigue and Its Impact on ADLs	Perceived fatigue is a major factor in daily life.	Perceived fatigue reduces abilities to perform ADLs. Perceived emotional/social fatigue impacts relationships and social lives.
	Perceived fatigue levels fluctuate. Perceived physical, cognitive/intellectual, and emotional/social fatigue are intertwined.	Patterns of perceived fatigue generally correlate with recent levels of physical, cognitive/intellectual, and emotional/social exertion. Perceived fatigue fluctuates in patterns that are unique to the individual. Depression and pain can increase perceived physical and emotional/social fatigue. Environmental conditions like cold can increase perceived physical fatigue. For some patients, treatment seems to have reduced physical fatigue or improved recovery from fatigue. Physical fatigue has the greatest impact on daily life. An increase in any one of the three types of perceived fatigue may cause an increase in one or both of the other types of perceived fatigue.
	Whether or not one talks to one’s doctor about perceived fatigue depends on the doctor and the patient.	All three types of perceived fatigue affect abilities to perform ADLs. Some people with SMA may not consider their doctor to be an expert in fatigue. Some people with SMA initiate conversations about perceived fatigue with their doctors to get medical help with or advice about managing fatigue. The topic of perceived fatigue sometimes comes up naturally during a visit to the doctor.

ADLs = activities of daily living; *n = 6

### Impact of perceived fatigue and perceived fatigability on ADLs

The second objective of the Non-ambulatory Discussion Group was to gather input from non-ambulatory adults with SMA about how perceived fatigue affects their abilities to perform ADLs. (Table 4) On a scale of 1–5 (1 = “hardly ever” and 5 = “all the time”), participants were asked to indicate the degree to which perceived fatigue is present in their everyday lives.^2^ Of the six Non-ambulatory Discussion Group participants, two answered “5,” one answered “4,” two answered “3,” and one selected “NA.”. Participants described how perceived fatigue levels fluctuated over time in patterns that were unique to the individual. One participant remarked that their energy was typically highest in the morning and decreased throughout the day. Another mentioned that their perceived physical fatigue varied throughout the day and week, but that they “bounced back” from fatigue more quickly since being on treatment. A third participant remarked that the weather greatly impacts their perceived fatigue because they have a fused spine, and their pain increases in cold weather, which in turn increases their fatigue. They explained that depression also notably increases their perceived fatigue, and that their fatigue decreases when the depression is successfully treated. Several other participants agreed that weather also affects their perceived fatigue levels. One participant explained that as they have gotten older and lost function, their perceived physical fatigue patterns have become very predictable: the busier their day, the more fatigue they experience.

But, 20 years ago, I experienced nothing even close to what I have now…I dread when people ask me to do anything past 5 o’clock because if they don’t have SMA, it’s embarrassing to tell them, “I’m just too tired.” I almost am so fatigued that I hurt. It’s almost like a pain, because it is so significant.

Non-ambulatory Discussion Group participants were then asked which type of perceived fatigue – physical, cognitive/intellectual, or emotional/social – is most troublesome. The majority ranked physical fatigue as having the greatest effect, but the group agreed that the different types are intertwined.

Sometimes my friendships kind of fall to the wayside because I’m just interacting with people all day, and asking for help, it physically and emotionally is exhausting.

If I didn’t have the physical fatigue, I would not have the social fatigue.

A third Non-ambulatory Discussion Group participant explained that it would be important for healthcare providers to be specific when inquiring about how perceived fatigue affects ADLs, because although their emotional fatigue affects their abilities to perform ADLs, the emotional fatigue is likely caused by depression rather than SMA.

Next, participants were asked, “How many of you have had the experience of talking either to your primary care doctor or specialist about fatigue?” The group reported mixed experiences.

I thought [the question] was funny. I’ve never had a conversation with my doctor about fatigue. I guess I just think it comes with the territory and what can they do about it?

If I were to feel the need to discuss [fatigue], probably one of the last people I would feel the need to discuss it with would be my doctor.

This participant explained that he liked his doctor but wouldn’t think of his doctor as being an expert on fatigue. However, a third participant had the opposite perspective:

I have a lot of different doctors, and I’ve talked to pretty much all of them about fatigue…Because in some way or another, it affects every specialty doctor that I see, for whatever reason that I see them.

Although the concept was not directly explored, one participant described a productive conversation they had when talking with their doctor about perceived fatigability. The participant told their physician that although they had received many physical accommodations when they began working full-time, they had not been given a long enough lunch break:

I was getting tired having to feed myself and rush eating during the 30 minutes. Plus, I didn’t have any downtime…I don’t think they realized that [eating] was also a physical activity.

In addition to providing an accommodation letter, the participant’s doctor discussed strategies for managing workplace fatigability to alleviate the need for reduced hours. Through referral to specialists, the participant was able to get both medical help and practical advice that improved their perceived fatigability in the workplace.

 At the end of the discussion session, participants were asked, “What do you wish your doctor understood about the fatigue experience?” Although there were no immediate responses, one participant eventually said:

…The fatigue is just so, it’s so debilitating for me, and it’s become that way more and more as I get older. And maybe part of that’s from, like, menopause, and that kind of thing but…it’s so terrible and devastating for me that it’s not so much what can I accomplish, but it’s how fatigue plays into that and…how it limits me.

### Ambulatory Discussion Group results

#### Activities of daily living (ADLs)

The first objective of the Ambulatory Discussion Group was to gather input from participants about which abilities to perform ADLs were most important to them to maintain. (Table 5) Ambulatory Discussion Group participants were asked to review a list of ADLs (Additional File 7) and indicate other ADLs of importance that were not included. Picking things up off the floor, emptying the dishwasher, using the toilet away from home, and running errands were noted as key ADLs by Ambulatory Discussion Group participants. Ambulatory Discussion Group participants also shared strategies and tools—like shower chairs, toilet doughnuts, and pinchers—they used to accomplish ADLs as motor abilities waned. Adaptation became a theme throughout the conversation. The group was asked which ADLs they were most concerned about retaining the ability to perform. ADLs related to maintaining independence and preserving dignity—such as driving, feeding oneself, and taking care of one’s own hygiene and appearance—were of importance.

To go wherever you want, whenever you want, and then the day that you know you can’t. It’s just, it, this disease, has robbed, robbed me. You know, I feel like, “Give me my life back.”

I think the shower, showering, bathing, toileting, you know, keeping clean, maintaining your appearance is probably on the top of the list for most of us. It’d be difficult to be at somebody else’s mercy.

Two Ambulatory Discussion Group participants had siblings with SMA, and the participants had watched their siblings lose abilities to perform ADLs related to preserving independence and dignity. These participants expressed concern about becoming similarly dependent on others.

Navigating new environments and environmental hazards inside and outside the home was also a theme of the discussion. Fear of falling, which could lead to a debilitating injury, was prominent for several of the participants.

It’s not on the list, but one thing that I see in daily living for me is the fear of falling. I live in fear of falling, because if I fall, I can break a hip…So, I might be overboard in it, but I look at every little thing. I’m always looking down for an acorn, a pebble, anything that’s uneven—a slight little thing that can cause you to fall. It’s starting to rule my life.

The participant went on to explain that they were careful to choose certain types of footwear and were selective about the type of chair they sat in to decrease their likelihood of falling.

Ambulatory Discussion Group participants were then asked, “How hard or easy do you think it would be to measure your overall health status based on your ability to perform ADLs over the course of time?” The moderator added that Non-ambulatory Discussion Group participants had shared that they often adapted methods of completing tasks to compensate for loss of function. One participant noted they own multiple identical assistive tools, such as “grabbers,” so that they can keep one in each room of the house. The same participant said that among other household adaptations, they had replaced their heavy dishware with lighter plates and cups.

*We figure out a way to do it*, *but we do things differently or we find things that work great for us.*

Another participant concurred, giving the example of having learned to break down the task of unloading the dishwasher into several discrete steps as she has lost muscle strength, rather than transferring dishes directly from the washer to the shelf. A third Ambulatory Discussion Group participant said,

*I look back over my life*, *and the whole thing is like an adaptation. Even before I knew I had SMA*, *I had different ways than other people of doing things because I had weakness…doing things over my head and things like that. And you just adapt here and adapt there and you figure out all these little cheats*, *so to speak. When they diagnosed me*, *I looked back and I thought*, “*Oh my gosh*, *now I know why everybody else does it like this. And I do it [like that].*”

The Ambulatory Discussion Group were then shown an example of a ranking scale that comprised the following choices: Unable – can’t do, doesn’t know how, or is too young; Hard – does with a lot of help, extra time or effort; A little hard – does with a little help, extra time, or effort; and Easy – does with not help, extra time or effort, or a person’s skills are past this level. The moderator asked, “If you are asked to rate your ability to perform various ADLs using a scale like this, would you find it easier to rate the broad domain like ‘feeding oneself,’ or would you want to be able to break the task into different parts?” Participants agreed that tasks broken down into smaller parts would be more likely to detect adaptations to accomplish ADLs as functional status changed.

**Table 5:** Summary of feedback from ambulatory discussion group participants*

Topic	Major theme	Subtheme
ADLs	Maintaining independence and preserving dignity are of the upmost importance.	Preserving abilities to perform activities like driving and toileting is very important.
	Adaptation is a key strategy for coping with loss of function.	As function declines, some tasks can still be performed but require different strategies, specific assistive tools, or help from others to accomplish. ADLs need to be detailed enough to detect adaptations that had occurred because of functional decline.
	Environmental conditions impact abilities to perform ADLs.	New environments with unknown accommodations and hazards are stressful. Falling inside and outside the home is a major concern—furniture, ice, uneven surfaces, and crowds are all potential threats to stability. Inclement weather reduces ability and willingness to participate in activities outside the home. Cold weather can also negatively affect mobility/dexterity and increase pain.
Perceived fatigue and Its Impact on ADLs	Some ADLs are related to caring for others. Perceived physical fatigue is an aspect of daily life.	Parents with SMA must adapt to perform parenting ADLs. Cooking for others as well as oneself may be an ADL for some people with SMA. Perceived physical fatigue levels are variable throughout the day. Patterns of perceived physical fatigue vary between participants.
	Physical fatigue is the most prominent type of perceived fatigue.	Physical fatigue has the greatest impact on ADLs and can precipitate perceived cognitive/intellectual and emotional/social fatigue.
		Environmental stressors like cold weather, wind, and obstacles can affect both perceived physical and perceived emotional/social fatigue.
	Healthcare professionals generally do not recognize that perceived fatigue is a component of SMA.	Healthcare professionals may be unfamiliar with SMA and attribute perceived fatigue to other causes. Even an SMA specialist may have a hard time appreciating the nature and level of a patient’s perceived fatigue.

ADLs = activities of daily living; *n = 4

### Impact of perceived fatigue on ADLs

The second objective of the discussion was to gather input from ambulatory adults with SMA about how fatigue affected their abilities to perform ADLs (Table 5). On a scale of 1–5 (1 = “hardly ever” and 5 = “all the time.”), Ambulatory Discussion Group participants were asked to indicate the degree to which perceived fatigue is present in their everyday lives.^3^ Two participants answered “5,” and two answered “NA.” Upon further discussion, it became clear that fatigue was a factor for each participant but presented in different forms and patterns for each individual. Whereas one participant felt very energized in the morning but “crashed” after their midday meal, another felt notable, painful fatigue throughout each day. A third participant’s fatigue fluctuated daily depending on sleep quality and the needs of their daughter. The fourth participant felt less fatigue in the afternoon than in the morning. Ambulatory Discussion Group participants were asked which of the following types of perceived fatigue were most troublesome: physical, cognitive/intellectual, or emotional/social. Participants unanimously agreed that perceived physical fatigue was the most troublesome. However, there was also discussion about how physical fatigue could precipitate perceived emotional/social fatigue. One participant explained,

I feel like my emotional and social fatigue is based on my physical fatigue. If I’m tired, then it’s physical. I don’t want to go and have to worry about somewhere not being accessible or being in a social situation and being down here when everybody’s up here—because I use my electric wheelchair most of the time.

When participants were asked if fatigue was something they talked to their doctors about, responses varied widely. One participant said they talked to all their doctors about fatigue and received different responses depending on the doctor. Another participant was evaluated for depression when they told their doctor they were experiencing fatigue. A third participant said that although their doctor is a specialist in SMA and will address fatigue by doing bloodwork and looking for ways to support them, they still hesitate to bring up the topic.

I think sometimes it’s hard. I don’t know if other people feel this way, but all my doctors at the neurology clinic, none of them have SMA, right? So sometimes it’s hard. It’s like, “Do I even attempt to explain?” Because they’re not going to understand it…So sometimes it’s like, “Is it even worth the effort to describe it or to communicate it?” Because I don’t think it’s ever going to be fully understood.

The moderator asked Ambulatory Discussion Group participants what they wished their doctor understood about the fatigue experience. Respondents broadly indicated they wished their doctor knew more about SMA, since the lack of familiarity impacts perceived ability to adequately care for SMA-related needs, and the physician’s ability to take the limitations of the disease into account when addressing needs unrelated to SMA. One participant noted their concerns are often attributed to their age, or even to menopause. Another participant remarked that their doctor had taken the time to educate himself about SMA:

I think the main thing is that when you do go to a doctor that they at least take the time to get the basic knowledge of [SMA]…to help us live our lives the best that we can. So, I guess it’s education, just educate themselves a little bit more.

Ambulatory Discussion Group participants were invited to share other factors that increased fatigue. Inclement weather, pain, and poor sleep quality were again mentioned as contributing factors. Conversely, one participant noted that treatment seemed to temporarily reduce their fatigue.

## Discussion

As more therapeutic options become accessible to teens and adults with SMA [68], it is important to establish a standardized set of core outcomes to measure disease progression and treatment response. Efforts to standardize outcome measures for teens and adults with SMA are underway in Canada and throughout Europe [44, 58, 69–72], but a core set of outcome measures has not yet been defined in the U.S. In the present study, we surveyed a working group of key opinion leaders in clinical research and care, as well as two discussion groups comprised of non-ambulatory adults or ambulatory adults with SMA, for their perspectives on what type of outcomes could capture disease status and treatment effects in a manner that is meaningful both clinically and to the individual. The study objective was to begin to build consensus toward a standardized set of outcome measures for teens and adults with SMA.

### Activities of daily living (ADLs)

The KOL Working Group reached consensus that ADLs are the best type of outcome to detect clinically relevant functional changes in teens and adults with SMA. KOL Working Group participants ranked ADLs that facilitate personal autonomy as most meaningful, and discussion group participants concurred that ADLs related to dignity and independence are of the upmost importance to maintain. The KOL Working Group and Discussion Groups also agreed that when used as outcomes, ADLs should be small, specific tasks selected to detect subtle changes in function that affect individuals’ unique lifestyles. Thus far, U.S. research studies assessing SMA disease progression or treatment effects in teens and adults have utilized predominantly CROMs such as the Hammersmith Functional Motor Scale Expanded (HFMSE), the 32-item Motor Function Measure (MFM32), the Revised Upper Limb Module (RULM), the 6 Minute Walk Test (6MWT); and the 10 Meter Walk/Run Test (10MWRT) [73–77]. A few studies have also included PROMs [78, 79], and several groups are actively developing novel CROMs [80] and PROMs [67], some of which were unpublished at the time of this writing. Whereas clinician-reported measures are utilized, by definition, in a clinical setting, PROMs have the potential to detect functional changes that are impactful to people with SMA as they go about their everyday lives. PROMs based on abilities to perform ADLs are commonly employed to assess disease status in adults with other neuromuscular diseases such as multiple sclerosis (MS) [81], amyotrophic lateral sclerosis (ALS) [82], and myasthenia gravis (MG) [83]. Since the present study was completed, several studies have also investigated the use of ADLs as outcomes to track disease progression and treatment response in teens and adults with SMA. For example, Sadjadi et al. (2023) [1] evaluated the modified Spinal Muscular Atrophy Functional Rating Scale (SMAFRS) as an ADL-based outcome measure for non-ambulatory and ambulatory adults, and Slayter et al. (2023) [2] included the SMAFRS in their eight-measure toolkit created to assess disease progression in adults. In addition, the Spinal Muscular Atrophy Independence Scale Upper Limb Module (SMAIS-ULM) has recently been developed as an ADL-based PROM for individuals who are 12 years of age or older, and for caregivers of children who are older than 2 years of age [84]. Finally, Lefton-Greif et al. (2025) [85] developed and partially validated a scale for bulbar function assessment in adults with SMA based in part on ADLs such as completing meals, swallowing pills, and communicating with family members. These studies demonstrate a trend in using outcome measures comprised of ADLs in assessing disease status and treatment response in teens and adults with SMA.

In Phase 1 of the study, the KOL Working Group did note that an outcome measure consisting of ADLs might be most powerful when combined with a clinical measure such as a biomarker. While recent research has found that molecular biomarkers reflecting neurodegeneration are not as useful in teens and adults with SMA as they are in children [65, 86, 87], electrophysiological outcome measures such as compound muscle action potential (CMAP) show promise as biomarkers in teens and adults with SMA [65, 70, 72, 74, 88]. Indeed, when asked to rank which type of outcome measure would best capture disease state in teens and adults with SMA, KOL Working Group participants ranked CMAP and ADLs as equally useful.

### Perceived fatigue and perceived fatigability

Several clinician-reported, functional outcome measures have been utilized to assess performance fatigability in teens and adults with SMA. For example, the 6MWT [89–93] has been the gold standard for quantifying lower limb performance fatigability in ambulatory individuals with SMA [37, 74, 94–97]. The Endurance Shuttle Nine Hole Peg Test (ESNHPT) has been used for distal arm and hand performance fatigability, and the Endurance Shuttle Box and Block Test (ESBBT) has been utilized to assess upper arm performance fatigability [98–100]. These and other clinician-reported, functional outcome measures of fatigability are advantageous in that they are objective. However, these assessments are not appropriate for the measurement of perceived fatigue and fatigability for several reasons. First, they measure the ability to perform specific assigned tasks, and these tasks may not be varied or sensitive enough to reveal subtle but important changes in perceived fatigue and fatigability that affect overall quality of life [101]. Second, people with very severe forms of SMA may not be able to complete standard functional tests, creating floor effects [102, 103]. Similarly, ceiling effects on existing functional assessments will likely become more salient as more people with SMA receive DMTs and other treatments, diversifying and broadening the natural history of the disease. Third, performance fatigability assessments are a snapshot of function in a clinic at a specific time point and are subject to variations based on recent activity and other variables. Finally, results from clinician-reported, performance fatigability outcome measures tend to correlate with disease severity and with results from functional assessments like the RULM and HFMSE [37, 104, 105]. In contrast, results from performance fatigability assessments have generally *not* correlated with levels of *perceived* fatigue assessed within the same studies. That is, improvements in performance fatigability do not correlate with improvements in perceived fatigue when the two are measured side-by-side [48, 101, 104]. Furthermore, levels of perceived fatigue generally do not correlate with disease severity or motor function [48, 59]. These findings suggest that the PROMs utilized in these studies may not have been sensitive enough to detect improvements in perceived fatigue. KOL Working Group and Discussion Group participants agreed that perceived fatigue and fatigability are components of everyday life that affect abilities to perform ADLs. Despite this agreement, participants in both Discussion Groups reported that most healthcare professionals do not recognize the significance of perceived fatigue and fatigability in the context of SMA. This communication gap may in part result from the fact that a PROM for perceived fatigue and fatigability in SMA had not yet been validated at the time of the study. Myriad PROMs have been used to assess perceived fatigue in teens and adults with SMA, such as the Fatigue Severity Scale (FSS) [95, 106], the Pediatric Quality of Life Multidimensional Fatigue Scale (PedsQL MFS) [48], the Multi-Dimensional Fatigue Inventory (MFI) [55], and the Spinal Muscular Atrophy Health Index (SMA-HI) sleep and fatigue modules [107]. These instruments query individuals about perceived levels of fatigue and fatigability experienced over durations ranging from the past week to the prior month. However, more recent research [57] has validated the use of a perceived fatigability scale comprised of ADLs. First, a new questionnaire, the PROfuture, was developed in Spain for people with SMA who are older than 14 years of age. Second, Rodriguez-Torres et al. (2025) [53] recently developed a novel PROM, the SMA EFFORT, for evaluating perceived physical fatigability in teens and adults across four categories: (1) exercise/recreation, (2) mobility, (3) ADLs, and (4) postural control. These new developments support the idea that ADLs can serve as outcomes for a standardized measure of perceived fatigue and fatigability in teens and adults with SMA living in the U.S.

### Limitations

During the Discussion Group phase of this study, the moderator did not delineate between perceived fatigue and perceived fatigability. In future surveys, the operational definitions of perceived fatigue and perceived fatigability should be introduced before people with SMA are asked to describe their lived experiences. Furthermore, the KOL Working Group represented perspectives from experts in SMA research and clinical care. Primary care physicians, physical therapists, neurologists, and other clinical care providers who are not as familiar with SMA may have contributed unique and valuable viewpoints to the study. Similarly, the Non-ambulatory and Ambulatory Discussion Groups were small and represented a limited range of demographics. In particular, the Discussion Groups did not include teen participants owing to lack of response during recruitment. Recruitment strategies for future surveys should be refined to encourage participation from teens living with SMA. Although limited by sample size, this study can serve as a starting point for a broader discussion on how to develop a core set of outcome measures for function, fatigue, and fatigability in teens and adults with SMA in the U.S.

## Conclusion

The findings of this study provide insight into which type of outcomes are meaningful to SMA researchers and clinicians, as well as adults with SMA, when assessing function, perceived fatigue, and perceived fatigability. The KOL Working Group and Discussion Groups agreed that PROMs based on ADLs may be most effective at detecting changes in disease progression, motor function, and perceived fatigue and perceived fatigability that impact abilities to perform everyday tasks that matter most to people with SMA. The groups also concurred that ADLs which impact one’s independence and dignity are of the highest priority to maintain and monitor. Furthermore, the KOL Working Group and Discussion Groups each acknowledged that perceived fatigue and fatigability are major aspects of daily life that affect abilities to perform ADLs, but that there is currently no effective way to measure or communicate about perceived fatigue and fatigability. Critical treatment needs remain unmet within the SMA community, particularly among teens and adults with SMA who do not receive DMT until later in disease progression. As novel treatment options and protocols become available for these populations, a standardized core set of outcome measures will be required to assess disease status and treatment response. These results may form a foundation for the development of core outcome measures for function, perceived fatigue, and perceived fatigability in teens and adults with SMA.

## Data Availability

All data generated or analyzed during this study are included in this published article and its supplementary information files.

## Footnotes

2 At the time the survey was conducted, operational definitions of the various aspects of fatigue and fatigability were not yet established. Therefore, Discussion Group participants were asked how “fatigue” affected their lives. In the prior Introductory Session, participants had been introduced to the concept of perceived physical, cognitive/intellectual, and emotional/social fatigue.

3 At the time the survey was conducted, operational definitions of the various aspects of fatigue and fatigability were not yet established. Therefore, Discussion Group participants were asked how “fatigue” affected their lives. In the prior Introductory Session, participants had been introduced to the concept of perceived physical, cognitive/intellectual, and emotional/social fatigue.
